# Puzzling relationship between levels of toxic metals in blood and serum levels of reproductive hormones: Benchmark dose approach in cross-sectional study

**DOI:** 10.1080/26895293.2022.2128439

**Published:** 2022-09-28

**Authors:** Ðurđica Marić, Katarina Baralić, Dragana Javorac, Stefan Mandić Rajčević, Danijela Ðukić-Ćosić, Evica Antonijević Miljaković, Miodrag Aćimović, Zorica Bulat, Michael Aschner, Aleksandra Buha Djordjevic

**Affiliations:** a Department of Toxicology ‘Akademik Danilo Soldatović’, University of Belgrade – Faculty of Pharmacy, Belgrade, Serbia;; b School of Public Health and Health Management and Institute of Social Medicine, Faculty of Medicine, University of Belgrade, Belgrade, Serbia;; c Clinic for Urology, Clinical Center of Serbia, Belgrade, Serbia;; d Albert Einstein College of Medicine, New York, NY, USA

**Keywords:** Toxic metals, reproductive hormones, Benchmark dose approach, endocrine disruptors

## Abstract

Reproductive disorders and infertility have become more common recently among the general population. Toxic metals are known as endocrine disruptors and as they are widespread in nature they may be linked to reproductive problems. This study was conducted as a cross-sectional study and its aim was to examine the dose–response relationship between cadmium, arsenic, mercury, chromium and nickel and serum hormone levels of testosterone (women) and estradiol and progesterone (men) using the Benchmark dose approach (BMD). Blood samples were collected from 218 women and 217 men digested in a microwave, and the levels of the tested metals were determined by atomic absorption spectrophotometry (AAS) or inductively coupled plasma-mass spectrometry (ICP-MS). Dose–response analysis was performed in PROAST software (version 70.1). The model averaging method was used to calculate the Benchmark dose interval (BMDI). A dose–response relationship has been established between all metals and hormones. The narrowest BMDI was found for the As-testosterone and Hg-testosterone. Levels estimated to produce the extra risk of testosterone serum levels disturbances of 10% were lower than median levels measured in the general population. Moreover, this research suggests the possibility of use of the BMD approach in analyzing data pool generated from extensive human-biomonitoring studies.

## Introduction

Throughout life, people are continuously exposed to a range of chemicals via different routes (oral, dermal, inhalation) and sources of exposure. Rather than being exposed to a single chemical, people are exposed to mixtures of substances, literally from the moment of conception and throughout life. Hence, their impact on health cannot be ignored (Hayes et al. 2019; Hernandez et al. 2019). It is now understood that humans simultaneously carry body burdens of numerous chemicals which have different properties and can exhibit a variety of effects (Nelms et al. 2018). Many of the chemicals we encounter daily can disrupt endocrine health, and they are defined as endocrine disruptors. These are exogenous substances or mixtures that change the functions of the endocrine system and consequently cause harmful effects on the health of the exposed organism, its offspring or (sub)population (WHO 2013). This group of chemicals includes several natural compounds (e.g. phytoestrogens), compounds used in the pharmaceutical industry, persistent organic pollutants, plasticizers, pesticides, industrial solvents but also toxic metals (Diamanti-Kandarakis et al. 2010; Yilmaz et al. 2020; Anlar et al. 2021). The topic of endocrine disruptors has been recently elucidated by Goumenou et al. (2021). The authors highlighted some enigmatic properties of this type of chemical, such as their ability to produce effects at low doses, the consequences of long-term exposure, non-monotonic dose–response, the (non)existence of a threshold, etc.

Toxic metals are normal constituents of the earth’s crust. However, as a consequence of primarily anthropogenic activity, they are emitted into the air, water, and food. Once released from the earth’s crust, these metals can remain in the environment for a long time because they are not subject to decomposition (Sparks 2005). According to the International Agency for Research on Cancer (IARC), metals such as chromium VI compounds (Cr), elemental arsenic and inorganic arsenic compounds (As), cadmium and cadmium compounds (Cd), and nickel compounds (Ni) are classified in the first group of carcinogens, while lead (Pb) is classified in group 2B, and mercury (Hg) and inorganic compounds of mercury are classified in the third group of carcinogens. The Agency for Toxic Substances and Disease Registry (ATSDR) has ranked As, Pb, Hg, and Cd as 1st, 2nd, 3rd, and 7th on the list of priority hazardous substances, respectively. This list is based on a combination of toxicity, frequency, and the possibility of exposure to the substances on the list.

Numerous studies have examined the relationship between exposure to environmental metals and the occurrence of various endocrine disorders. Metals such as Hg (He et al. 2013; Jia et al. 2021), Cd, Ni, and As (Chen et al. 2009) have been shown to be associated with the occurrence of diabetes mellitus. In addition, metals can affect insulin and glucose homeostasis, and it has been reported that As, Hg, Ni, and Cd may induce hyperglycemia, while Cr can contribute to the development of hypoglycemia (González-Villalva et al. 2016). It has also been established that toxic metals may have a harmful effect on thyroid function in occupationally exposed persons, while studies of the general population have been scant. In one such study that analyzed the results of the National Health and Nutrition Survey (NHANES) 2007–2008 in which the general population participated, elevated Cd levels were associated with decreased levels of thyroid-stimulating hormone (TSH), so thyroid function was impaired.

In recent years, an increased incidence of human infertility and reproductive health disorders has been observed with this trend expected to rise. Between 20% and 70% of the infertile population is men (Agarwal et al. 2015). Infertility can be linked to numerous risk factors (Chung et al. 2019; Ma et al. 2019; Bala et al. 2021). On that account, there have been studies that have the effects of toxic metals on reproductive health. Metals can disrupt the structure and function of cells in the male reproductive system. They lead to disorders of gametogenic cells, Leydig cells, and spermatogenesis (Chowdhury 2009; Tariba Lovaković 2020). The effect of metals on women’s reproductive function is often difficult to assess because factors such as age, ovarian reserve, and hormonal imbalance may significantly affect reproductive health. There are experimental studies linking exposure to toxic metals with problems such as miscarriage, fetal malformations, placental insufficiency, and premature birth together with changes in estrogen levels and even breast cancer occurrence (Sengupta et al. 2015; Anđelković et al. 2021). Most of the information on the impact of toxic metals on reproductive health, however, is derived from studies on experimental animals. In these studies, high doses of metals and short-term exposure are generally encountered, which fail to adequately represent environmental exposures (Pizent et al. 2012; Anđelković et al. 2021). Therefore, the advantage of applying data obtained in human studies is the lack of extrapolation of data to humans, but data analysis can often be complicated.

The Benchmark dose (BMD) concept makes it possible to establish a relationship between the dose of the test substance and the effect being monitored. This approach is considered more advanced compared to the No Observed Adverse Effect Level (NOAEL) approach. If the NOAEL approach was used to establish the dose–response relationship, the result would be the highest dose used in the experiment that does not lead to adverse effects. The disadvantage of this approach resides in the impact of experimental design on the results, single-point data, NOAEL dose variation between studies making it difficult to compare with other studies, and the inability to estimate the probability of response at any dose level. The main advantages of the BMD approach over the NOAEL approach include better data utilization, a wide range of data in relation to dose–response, and avoidance of the influence of the range of doses used in the study on the results. The result of the BMD approach is a dosing interval (not a single value) that is formed on the basis of a previously determined statistically significant change in effect (reference response – BMR) (Hardy et al. 2017). In addition to animal studies, the BMD concept can be applied to data obtained in human studies. The BMD approach is known to be used in the analysis of data obtained in experimental studies, but there is still little data on the use of this approach in epidemiological studies (Hardy et al. 2017). In addition, the European Food Safety Authority (EFSA) predicts the importance of applying the BMD approach in processing data obtained from epidemiological studies (Hardy et al. 2017). There are only a few epidemiological studies that use the BMD approach to interpret the results. An example of such a study is a study where it was necessary to obtain an appropriate dose of lead that could cause kidney damage. The concentration of lead in the blood was used as a biomarker of exposure, and the biomarkers of the effect were total proteins, beta (2) – microglobulin, and N-acetyl-beta-D-glucaminidase in urine. BMR was set to 10%, and data were processed as dichotomized in BMDS software (Lin et al. 2008). The number of studies that use the BMD approach in the analysis of data obtained from human samples is still insufficient, and thus far, only the previously described study by Lin et al has utilized such an experimental approach.

Our previous research reflected the effects of metals on testosterone levels in men. Narrow BMDI (0.273–3.62 μg Cd/L) was obtained for the Cd-testosterone ratio, which shows that a level of 0.273 μg Cd/L of blood can contribute to an increase in the risk of developing testosterone disorders in men by 10%. This is significant because the median concentration of Cd (1,476 μg /L) in the Serbian population is higher than 0.273 μg Cd/L (Baralić et al. 2022).

In this paper, we focused on the effect of metals on testosterone levels in women, and estradiol and progesterone in men. A small number of studies generally study the effects of metals on male sex hormones in women and female hormones in men, so we believe this paper would be a novel addition to the field. One of the reasons why we did not consider the effect of metals on estradiol and progesterone in women in this paper is the fact that the reference values for these hormones differ depending on the phase of the menstrual cycle. We did not have information on which subject was in which phase, so we could not know for sure whether the level of these hormones was disturbed or not. Although testosterone is known as the male sex hormone, and estradiol and progesterone are the female sex hormones, these hormones have significant functions in both sexes. Testosterone is important primarily given its role in anabolic, metabolic, and developmental processes in both men and women. Some of the consequences of testosterone disorders in women are irregular menstrual cycle, dysmenorrhea, hirsutism, polycystic ovary syndrome, etc. (Tyagi et al. 2017). The importance of testosterone for women is also highlighted by its potential treatment for infertility (Nagels et al. 2015). The importance of estradiol for the functioning of the hypothalamic-pituitary-testicular axis, growth hormone-insulin-like growth factor-1 axis regulation, bone growth, and glucose metabolism has been shown in men, but further study of the role of estradiol in men is necessary (Russell and Grossmann 2019). In men, the importance of progesterone is multiple – it affects spermatogenesis, and testosterone synthesis and is important in the diagnosis of prostate cancer (Oettel and Mukhopadhyay 2004). Considering the functions that these hormones have, a disorder of their levels can contribute to the damage to the reproductive health of both sexes.

Hence, the aim of this study was to analyze the dose–response relationship between measured metal concentrations (Cd, As, Hg, Cr, and Ni) and serum hormone levels: testosterone in women, estrogen, and progesterone in men relying on the BMD approach.

## Material and methods

### Study population

Blood samples from volunteers, representatives of the general population, were collected at the Clinical Center of Serbia and the Clinical Hospital Center ‘Bežanijska kosa’ in Belgrade, Serbia. 435 subjects participated in the study, out of which 183 subjects were healthy, and 252 subjects suffered from different diseases (breast cancer, prostate cancer, testicular cancer, pancreatic cancer, thyroid diseases). Among the respondents, there were 218 women and 217 men, between 18 and 94 years old. All study participants signed the informed consent. The study was conducted in accordance with the ethical guidelines defined by the Declaration of Helsinki. It was approved by the Scientific and Ethical Committee of the University Hospital Bežanijska kosa Medical Center (license number 9740/3), the Ethical Committee of the Clinical Center of Serbia (license numbers 526/9, 31/8, and 579/19), the Medical Faculty of the University of Belgrade (license number 1322 / XII-5) and the Ethical Commission for Biomedical Research of the University of Belgrade – Faculty of Pharmacy (license number 650/2 and 288/2).

### Sample preparation

Subjects’ blood was sampled after 12 h of fasting from the anterior cubital vein. Each sample is adequately marked with the identification number, date, and time of blood sampling. An aliquot of blood in Vacutainer tubes containing K2EDTA (BD Vacutainer^®^ system) was used to determine toxic metals. Blood was collected in additive-free tubes for serum separation. Serum was separated by centrifugation at 3000× g for 30 min after blood coagulation. Both EDTA blood and serum were kept frozen (at −20°C) until they were analyzed.

### Toxic metal analysis

Prior to analysis, samples were weighed (1 ml of EDTA blood) and digested in Teflon cuvettes by adding 7 ml of 65% nitric acid and 1 ml of 30% H_2_O_2_ in a microwave oven (Milestone START D, SK-10 T, Milestone Srl, Sorisole, Italy). Sample preparation was performed in three steps: heating for 15 min at 180°C, digestion for 15 min at 180°C, and cooling for 15 min. Also, blank samples containing a mixture of 65% nitric acid (7 ml) and 30% hydrogen peroxide (1 ml) were prepared and analyzed together with the samples. After cooling, samples and blanks were quantitatively transferred to normal 10 ml vessels. Graphite furnace atomic absorption spectrophotometry (AAS GTA 120 graphite tube atomizer, 200 series AA, Agilent technologies, Santa Clara, CA, USA) was used to determine the Cd concentration. The ICP-MS method (ICP-MS 7700, Agilent Technologies, Santa Clara, CA, USA) was used for the analysis of Hg, Cr, As, and Ni. An external standard technique (multielement standard solution 1 g/L in diluted nitric acid (Merck, Darmstadt, Germany)) was applied for calibration. The accuracy of both AAS and ICP-MS was validated with standard reference material (SRM) whole blood Level 2 (Seronorm TM, Sero, Billingstad, Norway). For SRM preparation and analysis, the same procedure was applied as for the EDTA-blood samples.

### Hormone analyses

The concentration of testosterone was determined in the serum of female subjects, while the concentration of estradiol and progesterone was determined in the serum of male subjects. Techniques used for hormone analysis are chemiluminescent immunoassay (CLIA) and electrochemiluminescent immunoassay (ECLIA) were used on the Liason (DiaSorin Inc, USA) and Cobas e411 analyzer families (Roche Ltd., Switzerland), with commercial reagents, according to good laboratory practices. A chemiluminescent reaction was performed with isoluminol derivatives with bovine serum albumin and a specific monoclonal antibody to the hormone that binds to metal particles. Streptavidin-coated microparticles with a specific anti-hormone monoclonal antibody are important for performing the electrochemiluminescent test. The resulting light was measured using photomultipliers as relative light units. A direct competitive chemiluminescent test was used to determine testosterone, estradiol, and progesterone.

### Dose–response modeling

Dose–response modeling was performed using PROAST software version 70.1 (the Dutch National Institute for Public Health and the Environment, RIVM). Data on testosterone, estradiol, and progesterone were analyzed as quantal individual data. The sex of the participants was used as a covariant. If hormone levels were in the reference range, they were assigned a value of 0, and if they were out of range, a value of 1. The reference values used for testosterone levels in women were 0.084–0.481 (20–49 years) and 0.029–0.408 (50 and several years) ng/ml. The reference values for estradiol concentration in men are 11.3–43.2 pg/ml, and for progesterone < 0.05–0.149 ng/ml. The model averaging method considers the results obtained by applying all available models and was used to calculate the BMD interval. The use of this approach has been recommended by the EFSA Scientific Committee because it adapts to model and data uncertainties (Hardy et al. 2017). The model was evaluated using the Akaike information criterion (AIC). This criterion shows how much the data fit into certain models and it is considered that the model with the lowest AIC value should be considered when defining the BMD interval (Hardy et al. 2017). Model averaging was performed using the bootstrap approach. For quantal data, the BMR is defined as a specified increase in incidence over background. A BMR of 10% extra risk was used. The BMD approach uses obtained curve to estimate the exposure level, where the extra risk is 10% together with the BMD confidence interval.

## Results

Table 1 shows the median, minimum and maximum values of the concentration of metals Cd, As, Hg, Cr and Ni in the blood of female and male subjects. The study examined the dose–response relationship between the concentration of Cd, As, Hg, Cr and Ni in the blood and the levels of testosterone in women, and estradiol and progesterone in the serum of men using the BMD approach. The bootstrap approach was used to average the model, and the measurement and evaluation of the model was performed using the AIC criteria. In the model averaging, the individual model results are combined using weights, with higher weights for models that fit the data better. Table 2 shows the models used in averaging, as well as the weights of each individual model.

Table 3 shows the BMDL (lower 95% confidence limit of the Benchmark dose) and BMDU (upper 95% confidence limit of the Benchmark dose) values obtained as a result of model averaging. The lowest BMDL for Cd is 8.19e-05 μg/L for the effect on estradiol concentration in men, for As is 5.79e-05 μg/L also for the effect on estradiol in men. The lowest BMDL for Cr is 7.49e-06 μg/L for the effect on testosterone in women. For Hg and Ni, the lowest BMDL values are 8.35e-06 μg/L and 2.02e-06 μg/L respectively for the effect on progesterone in men. In addition to BMDL values, the relationship between BMDU and BMDL or BMD interval (BMDI) is important for interpreting the results. The wider this interval, the more unreliable the result, and the width of the interval is affected by model and data uncertainties (Hardy et al. 2017). As the data are limited, the true BMD can never be defined exactly, and hence reporting a BMD confidence interval which is expected to include the true BMD with a defined level of confidence is provided. When the confidence interval is narrow (less than the factor 10 intervals), indicating high confidence in the estimates (Vieira Silva et al. 2022). However, the reliability and plausibility of the BMDL estimates do not have a direct impact on the model selection, therefore, some of the extreme lower and upper bound estimates are passed on to the best model for BMDL estimates. Hence, we have estimated that in this case, adequate BMDIs were obtained for As-testosterone and Hg-testosterone since although not 10-fold, they can be considered as relatively narrow.

**Figure 1.**
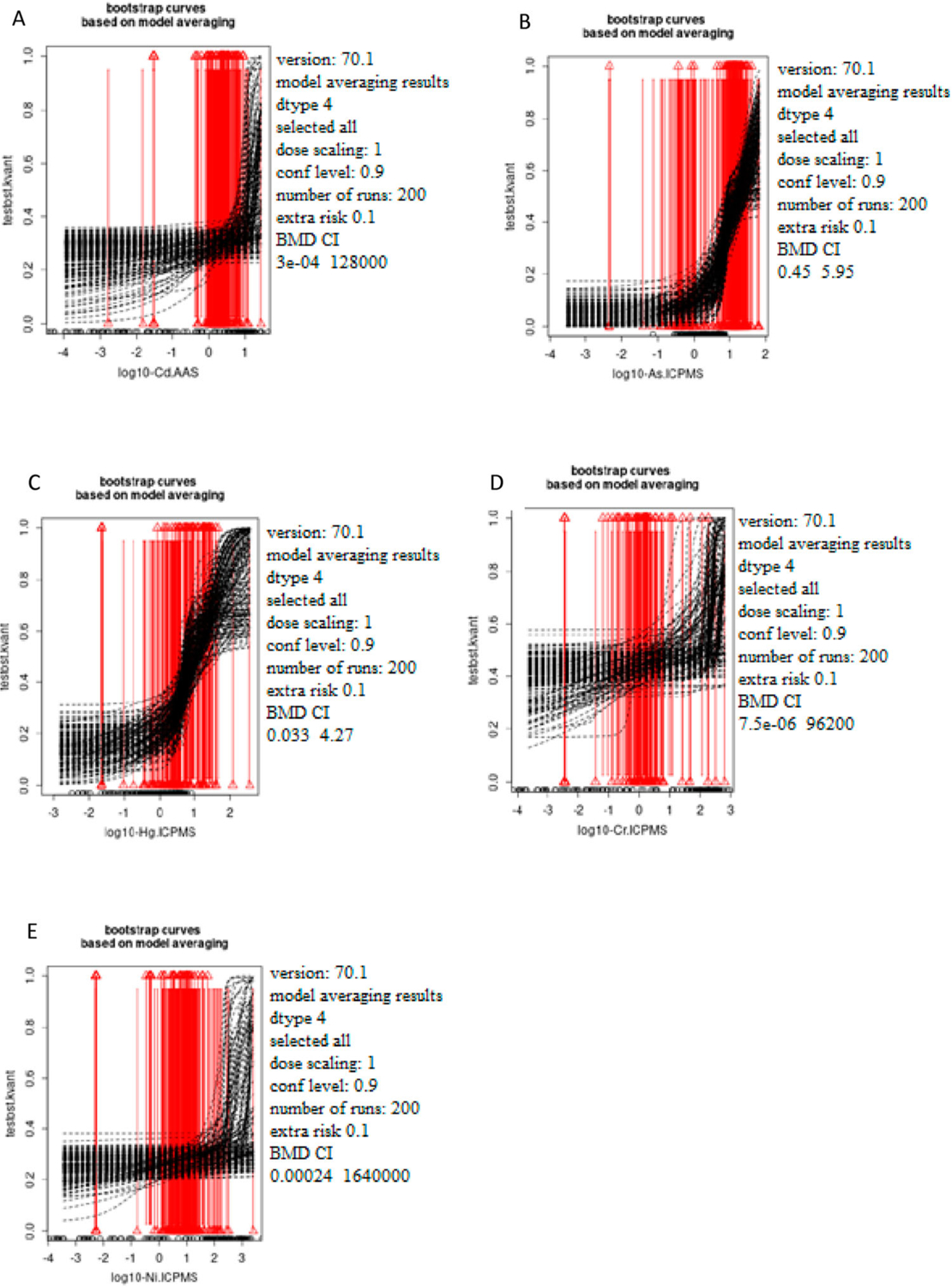
The dependence of the testosterone status (ng/ml) on the concentration of the selected metals Cd (A), As (B), Hg (C), Cr (D), and Ni (E) measured in women’s blood samples based on Model averaging. The x-axis represents log10 of Cd (A), As (B), Hg (C), Cr (D), and Ni (E) blood levels while the y-axis represents testosterone levels presented as quantal values (within/out of the range)

**Figure 2.**
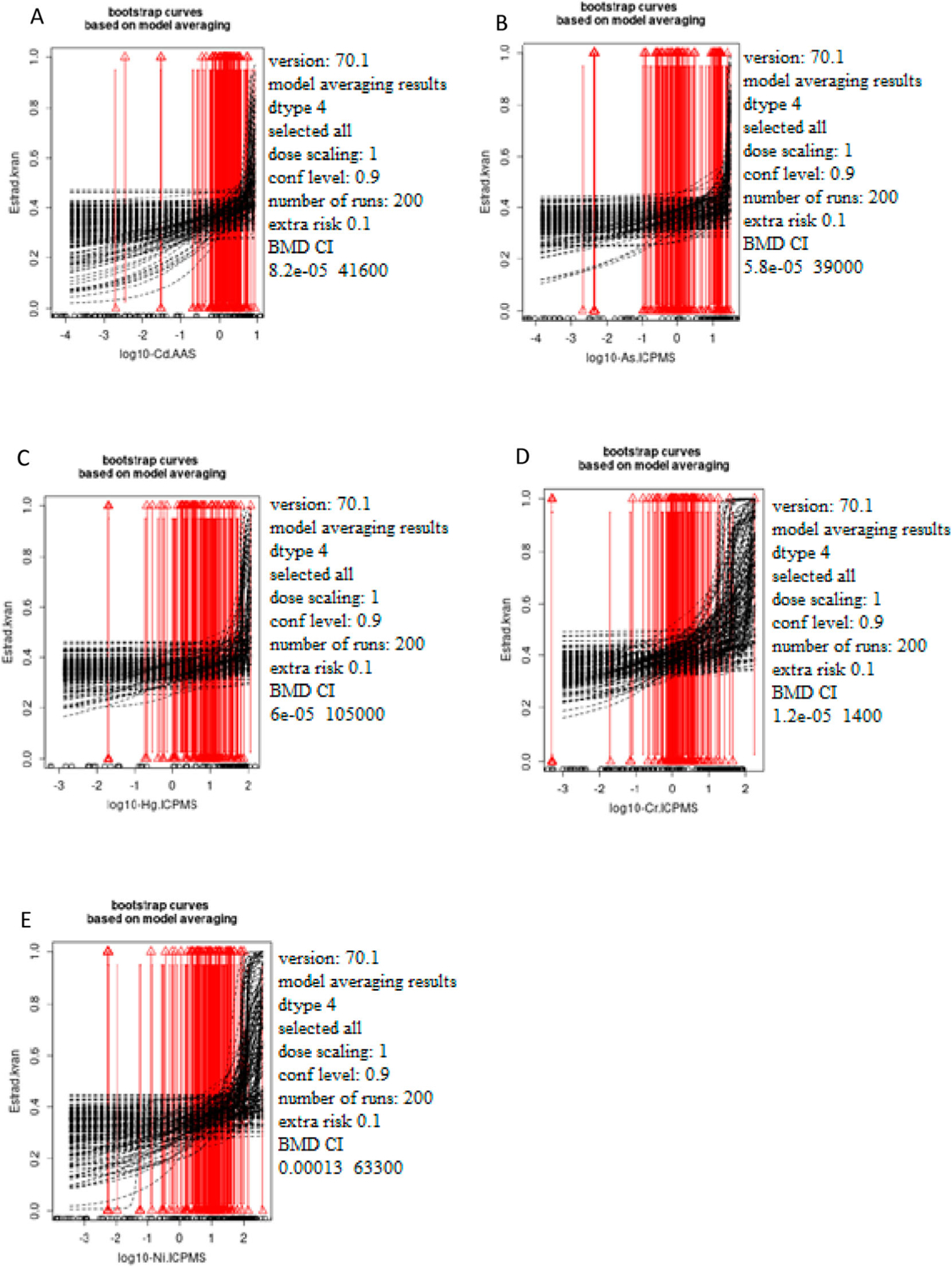
The dependence of the estradiol status (pg/ml) on the concentration of the selected metals Cd (A), As (B), Hg (C), Cr (D), and Ni (E) was measured in male blood samples. The *x*-axis represents log10 of Cd (A), As (B), Hg (C), Cr (D), and Ni (E) blood levels while the *y*-axis represents testosterone levels presented as quantal values (within/out of the range).

**Figure 3.**
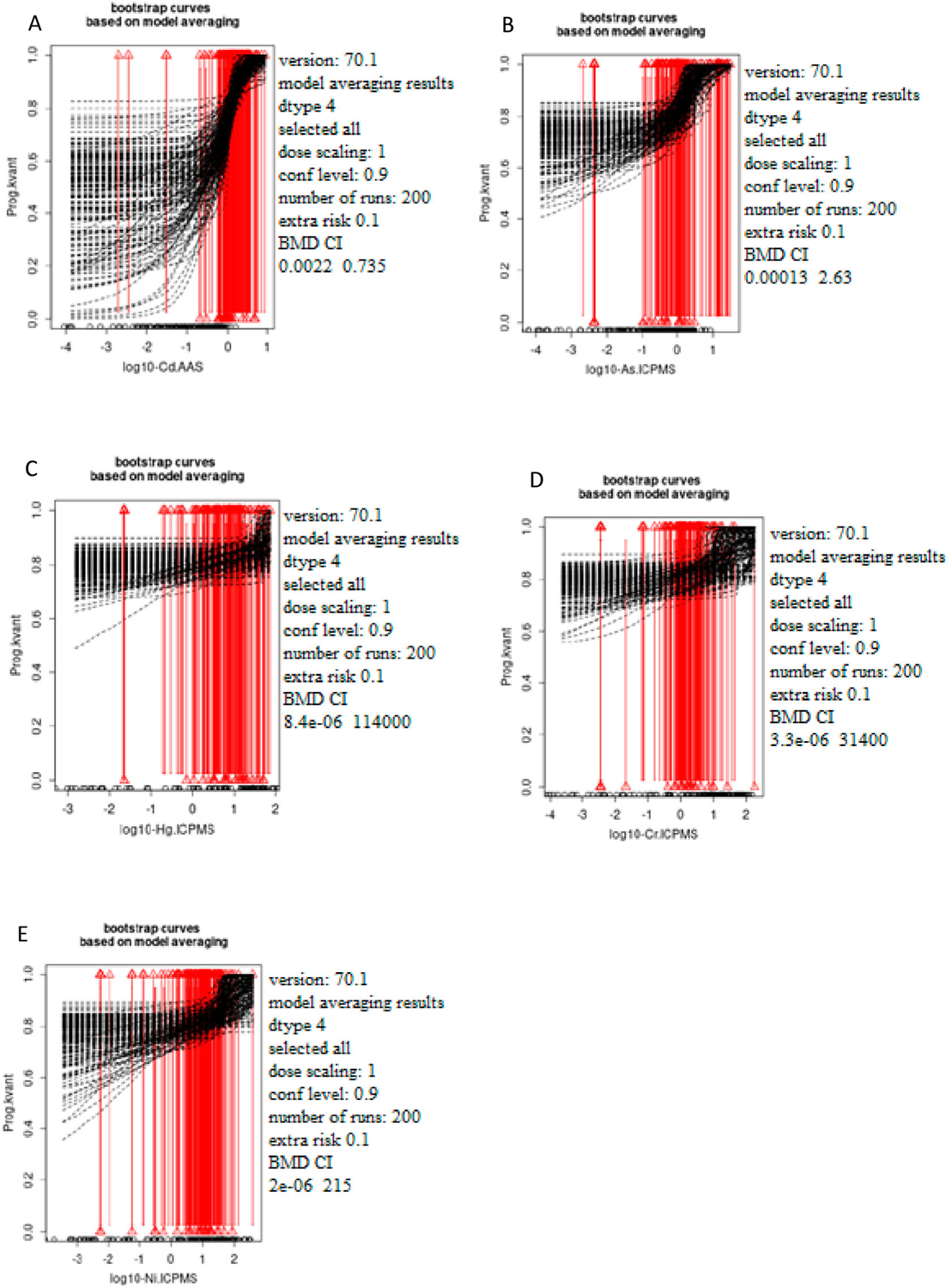
The dependence of the progesterone status (ng/ml) on the concentration of the selected metals Cd (A), As (B), Hg (C), Cr (D), and Ni (E) was measured in male blood samples. The *x*-axis represents log10 of Cd (A), As (B), Hg (C), Cr (D), and Ni (E) blood levels while the *y*-axis represents testosterone levels presented as quantal values (within/out of the range).

Figures 1–3 show the bootstrap curves (200 run) based on model averaging. Figure 1 shows the curves for testosterone, Figure 2 for estradiol, and Figure 3 for progesterone.

## Discussion

Various studies have addressed the effects of toxic metals on reproductive health. However, these studies are few, because of the difficulty in obtaining reliable information on the effects on the human population. Accordingly, most evidence obtained to date is derived from animal studies (Sengupta et al. 2015). The relationships between metals and hormones have been primarily analyzed by multivariable linear regression, which differs from the approach in this study. The PROAST software has been used to process data obtained in epidemiological studies. Using the BMD approach, it is possible to define the dose that leads to a change based on a previously determined statistically significant change in effect (reference response – BMR). The reference dose (BMD) refers to a response of 5% or 10%, and BMDL is the lower confidence limit for the selected BMD. The outcome of each BMD analysis is BMDI and not an individual value. In addition to BMDL values, the BMDU/BMDL ratio is important for interpreting the results of the analysis. Narrow BMDIs obtained for As-testosterone and Hg-testosterone indicate the greatest reliability of the obtained result. This implies that the levels of 0.45 μg As/L in the blood can increase the risk of testosterone blood level disorders in a woman by 10%. Conversely, the intervals obtained for Cd, Cr, and Ni were quite wide, indicating unreliability in the interpretation of the calculated values (Hardy et al. 2017).

It has been shown that several endocrine disruptors can affect the level of testosterone in the blood of experimental female animals, but also estrogen and progesterone in male animals. An example of one such substance is triclosan. Pregnant female rats were exposed to triclosan at a dose of 30–600 mg/kg b.w./day with oral gavage, and this exposure was associated with a decrease in the levels of more sex hormones, including testosterone (Feng et al. 2016). A study conducted by Castellanos et al. showed that an insecticide, specifically DDT, can disrupt estrogen and progesterone levels in the neonatal porcine Leydig cells (Castellanos et al. 2013). The effect of toxic metals on the disruption of testosterone levels in women, and estrogen and progesterone in men is still not sufficiently known. Therefore, the results of studies investigating this topic are quite limited. An earlier study in animals suggested that exposure to Cd may contribute to a reduction in plasma testosterone levels by having Cd act directly on ovarian steroidogenesis. It has been assumed that the effect depends on the dose, thus, low doses stimulate ovarian biosynthesis, while high doses have an inhibitory effect (Tomei et al. 2008). Given the importance of these hormones for the normal reproductive health of male and female populations, further research in this area is necessary.

As causes significant negative effects on the male reproductive system. The toxic effects of As are mainly related to oxidative stress and consequent damage to DNA and lipid membranes (Tariba Lovaković 2020). Due to a large amount of As that accumulates in the testicular tissue, it has been assumed that As readily crosses the blood-testicular barrier and directly exerts toxic effects on the tissue. In experimental studies, it has been observed that As alters testicular mass, and reduces sperm and sperm production. It is thought that As may also alter androgen receptor expression (Tariba Lovaković 2020). In addition, As negatively affects female reproductive function. A recent study has shown a link between high levels of As in the urine and an increased risk of developing primary ovarian failure, as well as direct effects of As on the level of reproductive hormones. In this case–control study, which comprised 169 women with primary ovarian failure and 209 healthy women, the authors monitored follicle-stimulating hormone (FSH), luteinizing hormone (LH), anti-Mullerian hormone (AMH), and estradiol. The relationship between As and ovarian insufficiency was explained by the fact that As exposure disrupted the synthesis of sex hormones and follicle development, leading to decreased number of ova and loss of ovarian activity (Pan et al. 2020). A study conducted on mice showed that As disrupted the level of estrogen hormones, in turn, causing preneoplastic changes in the testicles of mice (Guvvala et al. 2016). In a study conducted by Jana et al. male Sprague–Dawley rats were exposed to As via drinking water (5 mg/kg b.w./day) for 28 days. Certain groups received additional estradiol (25 micrograms oestradiol 3-benzoate suspended in 0.25 ml olive oil/day) or human chorionic gonadotrophin (5 I.U./kg body weight/day). Chronic exposure to As (III), in addition to leading to a decrease in testicular weight, also led to decreased synthesis of testosterone and gonadotropins and increased the activity of the adrenal cortex. Many of the observed side effects of As may be more prevalent under the influence of estradiol. It has been assumed that arsenite may act as an estrogen analog (Jana et al. 2006). There are no experimental data on the effect of As on testosterone levels in women, and the result of our study suggests that As levels higher than 0.45 μg/L may increase the risk of testosterone levels disturbances disorders by 10%. The median level of As in the blood of women from Serbia is 10.44 μg/L (Table 1), indicating the possible effects of this metal on testosterone homeostasis in women.

Several studies have examined the impact of Hg on women’s reproductive health. Consequences of Hg accumulation in the ovaries include altered reproductive behavior, infertility, and impaired ovarian function. Frequent occurrence of menstrual and hormonal disorders has been posited to be associated with Hg exposure (Henriques et al. 2019). Mercury has an inhibitory effect on the secretion of FSH and LH, thus increasing estrogen and progesterone levels in the body leading to painful menstruation, premature menopause, and menstrual cycle disorders (Davis et al. 2001). Some studies have examined the link between reduced human fertility and blood Hg levels. It has been shown that the consumption of seafood, coffee, alcohol, and smoking (which are the main sources of Hg) contribute to increased levels of Hg in the body and that with increased consumption of these products there is an elevated concentration of Hg in the body of infertile people. Namely, in people with infertility problems, a higher level of Hg in the body was observed compared to fertile individuals (Choy et al. 2002). Experimental studies have shown that the negative effects on the reproduction of male rats are manifested through impaired spermatogenesis, reduced sperm motility, and the occurrence of pathological changes (Kalender et al. 2013; Adelakun et al. 2020). A study conducted by Kalender et al. examined the effect of mercuric chloride on antioxidant status and histopathological changes in Wistar rat testis tissue. The results of the study indicated that mercuric chloride decreased the activity of several testicular enzymes in rats, such as superoxide dismutase (SOD), catalase (CAT), and glutathione peroxidase (GPx) compared to the control group. Changes in histology were observed in the treated compared to the control groups (Kalender et al. 2013). One study reported a negative effect of HgCl_2_ on the testes in male Wistar rats. After 28 days of oral administration of 40 mg/kg/b.w./day, significant impairment in testicular structure and function was observed. A significant reduction in sperm count and motility was also observed (Adelakun et al. 2020). The result of our study suggests that Hg levels greater than 0.033 μg/L may increase the risk of the development of testosterone levels disturbances by 10%. The median level of Hg in the blood of women from Serbia is 3.44 μg/L, implying that the female population might have an increased risk of testosterone levels disturbances due to Hg exposure.

Cd is known to have negative effects on the male and female reproductive systems. The mechanisms of Cd toxicity were investigated *in vitro* and *in vivo*. Its mechanism of toxicity includes the generation of reactive oxygen species (ROS). Peroxidation of cell membrane lipids and reduction of adenosine triphosphate levels may occur, which in turn reduce sperm motility (Wang et al. 2016). Cd can indirectly disrupt spermatogenesis due to interaction with bioelements (zinc (Zn), magnesium (Mg), iron (Fe), copper (Cu), calcium (Ca)) and the effect on their concentration in testicular tissue (Mouro et al. 2020). Due to the accumulation of Cd in the testicular tissue, Leydig cells can be damaged and testosterone production can be disrupted (Rana 2014). In addition, Cd can bind to estrogen and androgen receptors, and thus disrupt the function of sex hormones (Silva et al. 2012; Rana 2014). Monitoring testosterone disorders is also important in women as high testosterone levels have been linked to polycystic ovary syndrome, genital cancer, and altered puberty. Several studies have shown a link between Cd exposure and testosterone levels in men and women (Yao et al. 2019). A study conducted by Nagata et al. found a positive correlation between urine Cd levels and testosterone levels in the serum of Japanese postmenopausal women. Testosterone levels were significantly higher in women who had high levels of Cd in their urine (≥3 μg / g creatinine), in contrast to women who had low urinary Cd levels (< 2 μg / g creatinine). The disadvantage of this study is that the effect of duration of Cd exposure and the effect on hormones could not be determined. In addition, one should take into account the fact that the level of testosterone in circulation is higher in postmenopausal women and has a great impact on the body. It is believed that testosterone in women can contribute to depression, osteoporosis, diabetes, and coronary heart disease (Nagata et al. 2005). Also, Kresovich et al. found a positive relationship between the concentration of Cd in the blood and the levels of sex hormone-binding globulin (SHBG) (Kresovich et al. 2015). Our recent study revealed the dose–response relationship between Cd levels in blood and breast tissue and estrogen blood levels in women using the BMD approach (Buha-Ðorđević et al. 2021). An animal study has shown a significant role of progesterone in fetal development. It has also been shown that Cd can significantly contribute to limiting fetal growth in part and through inhibition of progesterone synthesis from the placenta in late pregnancy (Xiong et al. 2020). Chromium (VI), due to its instability, causes mutagenic and carcinogenic effects on cells. It leads to disorders of the male reproductive system through the formation of increased oxidative stress. In the testes, it negatively affects spermatogenesis because it acts by causing chromatid cleavage, damaging mitochondria, and disrupting the function of the blood-testis barrier (Aruldhas et al. 2005). Chromium also affects the function of antioxidants present in the testes. The negative effect of Cr primarily leads to reduced sperm quality and male infertility (El-Demerdash et al. 2019). In one study, pregnant female Sprague Dawley rats received doses of 0, 3, 6, and 12 mg/kg Cr (VI) as potassium dichromate. The testicles of the pups were collected and the number and function of Leydig and Sertoli cells were examined. A dose of 3 mg/kg Cr (VI) increased testosterone levels and did not adversely affect Leydig and Sertoli cells, while a dose of 12 mg/kg Cr (VI) impaired testicular function and decreased testosterone in rat blood (Zheng et al. 2018). In this study, it was shown that the manifestation of harmful effects in offspring occurs due to exposure to Cr *in utero*. Nickel can cause fertility disorders, abortions, and malformations. Nickel crosses the placenta and causes teratogenicity and embryotoxicity. Nickel influences the secretion of sex hormones such as FSH, LH, estradiol, and testosterone (Boulila and Ounassa 2020). Nickel nanoparticles have been shown to reduce estradiol levels when administered to female rats at doses of 15 and 45 mg/kg. In the same study, a drop in testosterone levels was observed (Kong et al. 2014). Gan et al. showed that after the application of Ni at a dose of 5 mg/kg, there was a decrease in the levels of testosterone and enzymes for its synthesis (Gan et al. 2019). However, the results of our study revealed wide BMDI intervals for these metals. Accordingly, further studies are needed to provide a more reliable conclusion regarding the levels at which these metals influence investigated sex hormones.

## Conclusion

Cd, As, Hg, Cr, and Ni have been linked to changes in testosterone levels in women and estrogen and progesterone levels in men. A dose–response relationship was established between all metals and hormones, however, narrow BMDI pointing to reliable data was found for As-testosterone and Hg-testosterone dose–response curves. The BMD approach estimated the levels where the extra risk for testosterone serum levels disturbances is 10%, and these levels were lower than median levels measured in the general population. However, studies utilizing appropriate animal models treated with appropriately defined low doses reflecting levels measured in humans will follow to establish the precise effects and mechanisms of such low doses of selected metals on the endocrine system. Moreover, this research suggests the possibility of further use of the BMD approach to analyze big data pools generated from extensive human-biomonitoring studies permitting significant refinement of the use of *in vivo* experimental data.

## Figures and Tables

**Table 1.** Median, minimum, and maximum levels of Cd, As, Hg, Cr and Ni in the blood of women and men who participated in the study.

Metal concentration		Cd (μg/L)	As (μg/L)	Hg (μg/L)	Cr (μg/L)	Ni (μg/L)
Women	Median	1.96	10.44	3.44	1.06	8.28
	Minimum	0.00	0.00	0.02	0.00	0.01
	Maximum	27.20	65.07	361.08	635.15	2532.54
Men	Median	1.48	0.97	5.22	1.32	7.61
	Minimum	0.00	0.00	0.02	0.00	0.01
	Maximum	14.65	34.55	118.15	765.49	381.30

**Table 2.** Models used for averaging and model weights.

	Model	Cd	As	Hg	Cr	Ni
Testosterone	two.stage	0.15	0.00	0.00	0.11	0.13
	log.logist	0.14	0.00	0.00	0.11	0.14
	Weibull	0.14	0.00	0.00	0.11	0.14
	log.prob	0.14	0.01	0.00	0.11	0.14
	Gamma	0.14	0.00	0.00	0.24	0.14
	EXP	0.15	0.72	0.51	0.14	0.14
	HILL	0.15	0.27	0.49	0.18	0.14
Estradiol	two.stage	0.14	0.19	0.19	0.09	0.00
	log.logist	0.14	0.02	0.02	0.18	0.07
	Weibull	0.14	0.19	0.19	0.18	0.21
	log.prob	0.14	0.03	0.26	0.18	0.09
	Gamma	0.14	0.19	0.19	0.18	0.21
	EXP	0.14	0.19	0.15	0.09	0.21
	HILL	0.14	0.19	0.19	0.10	0.21
Progesterone	two.stage	0.00	0.13	0.14	0.13	0.19
	log.logist	0.02	0.10	0.14	0.15	0.01
	Weibull	0.00	0.15	0.14	0.15	0.19
	log.prob	0.01	0.13	0.14	0.16	0.01
	Gamma	0.00	0.16	0.14	0.15	0.11
	EXP	0.86	0.16	0.14	0.13	0.19
	HILL	0.09	0.16	0.14	0.14	0.19

**Table 3.** Benchmark dose confidence intervals BMDI (BMDL–BMDU) for the selected metals (Cd, As, Hg, Cr, Ni) and hormone concentration.

	Testosterone		Estradiol		Progesterone	
Metal	BMDL	BMDU	BMDL	BMDU	BMDL	BMDU
Cd (μg/L)	0.000301	128,000	8.19e-05	41,600	0.00218	0.735
As (μg/L)	**0.45**	**5.95**	5.79e-05	39,000	0.000133	2.63
Hg (μg/L)	**0.033**	**4.27**	6e-05	105,000	8.35e-06	114,000
Cr (μg/L)	7.49e-06	96,200	1.19e-05	1400	3.32e-06	31,400
Ni (μg/L)	0.000235	1,640,000	0.000126	63,300	2.02e-06	215

## Data Availability

The data that support the findings of this study are available at https://data.mendeley.com/datasets/zbbgmvnz2r/3.
